# Modulation of adrenocorticotrophin hormone (ACTH)-induced expression of stress-related genes by PUFA in inter-renal cells from European sea bass (*Dicentrarchus labrax*)

**DOI:** 10.1017/jns.2015.6

**Published:** 2015-05-04

**Authors:** Daniel Montero, Genciana Terova, Simona Rimoldi, Lluis Tort, Davinia Negrin, María Jesús Zamorano, Marisol Izquierdo

**Affiliations:** 1 Universidad de Las Palmas de Gran Canaria (ULPGC), Grupo de Investigación en acuicultura (GIA), Instituto Universitario de Sanidad Animal y Seguridad Alimentaria (IUSA), c/ Transmontaña, s/n, 35413, Arucas, Las Palmas, Canary Islands, Spain; 2 University of Insubria, Department of Biotechnology and Life Sciences, Via Dunant, 3-21100 Varese, Italy; 3 Universitat Autonoma de Barcelona, Department de Biologia Cel.lular, Fisiologia i immunologia, Edifici M. 08193, Bellaterra, Cerdanyola del Vallès, Barcelona, Spain

**Keywords:** Stress-related gene expression, Fatty acids, Adrenocorticotrophin hormone-induced stress response, Nutritional modulation of steroidogenesis, *Dicentrarchus labrax*, ACTH, adrenocorticotrophin hormone, ALA, α-linolenic acid, ARA, arachidonic acid, COX, cyclo-oxygenase, CYP11b, cytochrome P450 11β, GR, glucocorticoid receptor, HSP, heat shock protein, LA, linoleic acid, LOX, lipo-oxygenase, PKA, protein kinase A, PLA2, phospholipase A2, StAR, steroidogenic acute regulatory protein

## Abstract

Dietary fatty acids have been shown to exert a clear effect on the stress response, modulating the release of cortisol. The role of fatty acids on the expression of steroidogenic genes has been described in mammals, but little is known in fish. The effect of different fatty acids on the release of cortisol and expression of stress-related genes of European sea bass (*Dicentrarchus labrax*) head kidney, induced by a pulse of adenocorticotrophin hormone (ACTH), was studied. Tissue was maintained in superfusion with 60 min of incubation with EPA, DHA, arachidonic acid (ARA), linoleic acid or α-linolenic acid (ALA) during 490 min. Cortisol was measured by RIA. The quantification of stress-related genes transcripts was conducted by One-Step TaqMan real-time RT-PCR. There was an effect of the type of fatty acid on the ACTH-induced release of cortisol, values from ALA treatment being elevated within all of the experimental period. The expression of some steroidogenic genes, such as the steroidogenic acute regulatory protein (StAR) and *c*-*fos*, were affected by fatty acids, ALA increasing the expression of StAR after 1 h of ACTH stimulation whereas DHA, ARA and ALA increased the expression of *c*-*fos* after 20 min. ARA increased expression of the 11β-hydroxylase gene. Expression of heat shock protein 70 (HSP70) was increased in all the experimental treatments except for ARA. Results corroborate previous studies of the effect of different fatty acids on the release of cortisol in marine fish and demonstrate that those effects are mediated by alteration of the expression of steroidogenic genes.

Cortisol, which is the main corticosteroid in fish^(^^1^^)^, is released into the bloodstream from the inter-renal cells under the action of adrenocorticotrophin hormone (ACTH) via activation of the hypothalamus–pituitary–inter-renal axis following a stressful situation^(^^2^^)^. ACTH stimulation of cortisol synthesis is mainly dependent on the cAMP/protein kinase A (cAMP/PKA) pathway, which involves a signalling cascade integrating G-proteins, cAMP and PKA^(^^3^^)^. However, there are other pathways described in fish^(^^4^^)^, independent of cAMP activation, including protein kinase C activation via stimulation of angiotensin II or acetylcholine with a final activation of genes involved in steroidogenesis, such as steroidogenic acute regulatory protein (StAR). StAR is involved in the transport of cholesterol through the mitochondrial membrane of the steroidogenic cells to be used as a substrate for steroid synthesis^(^^3^^)^. However, although cAMP is the most important second messenger for trophic hormone-stimulated steroid biosynthesis, other mechanisms independent of cAMP have been described, including macrophage-derived factors, and intracellular Ca and/or Cl ions^(^^3^^)^. Once the cholesterol is transported into the mitochondria, a cascade of enzymes, such as those belonging to the cytochrome P450 family, is activated. Cytochrome P450 11β (CYP11b) catalyses the last step that transforms 11-desoxicortisol to cortisol^(^^5^^)^ which is then released into the bloodstream following a stressful situation.

It has been reported that the use of vegetable oils in fish diets alters the post-stress circulating levels of plasma cortisol, both *in vivo*^(^^6^^–^^8^^)^ and *in vitro*^(^^9^^)^. The limited availability of fish oil to fulfil the increased demand in aquafeeds has induced the necessity to replace this oil by other oils, of marine or terrestrial origin^(^^10^^)^. Among the different oils used in the aquafeeds industry for such replacement, single vegetable oils or their blends seem to be good candidates that are being used in the diet of different fish species^(^^11^^)^. Fish growth is not affected by 60–75 % replacement of fish oil with alternative lipid sources, if essential fatty acids requirements are fulfilled. However, high levels of fish oil replacement can induce negative effects in marine fish, depending on fish size, water temperature, the type of oil used, and the amount of fish meal used in the diet^(^^12^^)^.

Vegetable oils are abundant in *n*-6 and *n*-9 C18 long-chain PUFA, mainly linoleic acid (18 : 2*n*-6, LA) and α-linolenic acid (18 : 3*n*-3, ALA), but are poor sources of long-chain PUFA, including EPA (20 : 5*n*-3), DHA (22 : 6*n*-3) and arachidonic acid (ARA; 20 : 4*n*-6), which are essential for marine fish. Although vegetable oils have been used in diets for marine fish species^(^^11^^,^^12^^)^, the reduction in the health-promoting effects provided by long-chain PUFA can be induced if a non-well-balanced blend of oils is used^(^^11^^)^. Indeed, the use of certain vegetable oils has been reported to alter different immune system-related parameters^(^^13^^,^^14^^)^ and to affect also the stress response in different marine species^(^^6^^,^^15^^)^. In particular, ALA has been shown to increase the *in vitro* release of cortisol from gilthead sea bream inter-renal cells, whereas LA produced the same effect but delayed in time^(^^9^^)^. As for the different essential fatty acids, the deficiency of *n*-3 highly unsaturated fatty acids has been shown to alter the post-stress plasma cortisol levels in gilthead sea bream^(^^16^^)^ and ARA has been reported to affect whole-body cortisol levels in larval stages of this species^(^^17^^–^^20^^)^.

However, the specific mechanisms involved in the modulation of the cortisol release by different fatty acids are still poorly understood. Ganga and *et al.*^(^^9^^,^^21^^)^ demonstrated that the role of different fatty acids in the release of cortisol from the anterior kidney is mediated, at least in part, by the action of cyclo-oxygenase (COX) and lipo-oxygenase (LOX) metabolites^(^^9^^,^^21^^)^, in a cAMP-dependent manner. However, these authors suggested mechanisms other than COX and LOX metabolites, in which certain fatty acids, such as DHA, modulate the release of cortisol from the inter-renal cells^(^^21^^)^, but they did not define such mechanisms.

In more recent studies, it has been shown that dietary fatty acids are able to modulate the expression of stress response-related genes in different marine fish species^(^^20^^,^^22^^)^, as it occurs in mammals^(^^23^^–^^25^^)^. The long-chain PUFA have been shown to down-regulate the expression of genes involved in the release of cortisol, such as StAR. Wang *et al.*^(^^25^^)^ described the role of ARA and epoxygenase metabolites from ARA in cAMP-stimulated steroidogenesis and in the expression of the StAR gene in MA-10 mouse Leydig cells. ARA regulation of steroidogenesis has also been described to be mediated by 5-LOX metabolites^(^^26^^)^. Wang *et al.*^(^^24^^)^ also described an effect of COX-2-derived ARA metabolites in steroidogenesis through StAR gene expression. C18 fatty acids have been shown to alter adrenal steroidogenesis both *in vivo* and *in vitro*^(^^27^^,^^28^^)^. In fish, the role of dietary lipids on StAR expression remains unclear. Only some effects of ARA in Senegalese sole whole post-larval StAR gene expression have been described^(^^20^^)^, without finding a clear correlation between dietary levels of ARA and the expression of this steroidogenic gene.

The regulation of steroidogenesis has been also linked to the action of the activator protein-1 (AP-1) family member c-*fos*^(^^3^^,^^29^^)^. The c-*fos* gene is well known as an immediate early gene because it is rapidly expressed in several mammalian brain sites in response to various stressful stimuli, including CO_2_/H^+^ elevation^(^^30^^)^. The product of this gene, the c-fos protein, is a nuclear factor that regulates gene transcription by binding to AP-1 regulatory elements in the promoter and enhancer regions of numerous genes^(^^31^^)^. Its biochemical characteristics and molecular nature have been widely studied; however, most of the research has been done using mammalian model species. Indeed, studies on c-*fos* expression under hypercapnic stress conditions have been carried out in mice^(^^32^^)^ and rats^(^^33^^,^^34^^)^. In both species, following CO_2_ stimulation, the expression of the c-*fos* gene was induced within minutes. In fish, the c-*fos* gene has been cloned in some species, such as *Tetraodon negroviridis*, *Carassius auratus*, *Ctenopharyngodon idella*, *Oncorhynchus mykiss*, *Rivulus marmoratus* and *Dicentrarchus labrax*^(^^35^^–^^38^^)^, but, to our knowledge, there is no information on tissue expression patterns of the c-*fos* gene related to different dietary fatty acids and stressful conditions in fish.

Cortisol effects in the cell are mediated by the glucocorticoid receptors (GR), which are members of the nuclear receptor superfamily that act as ligand-dependent transcription factors^(^^39^^)^. Within the cytosol, GR is present in a non-activated form together with heat shock proteins (HSP) such as HSP70 and HSP90, whose functions are the assembly, functionality and transport of GR^(^^40^^)^. HSP are associated to the GR until a hormone signal, such as cortisol, induces a conformation with lower affinity for HSP, dissociating GR from the HSP. Then, the receptors translocate into the nucleus and bind to a specific DNA region, the glucocorticoid response element, to regulate the transcription of glucocorticoid-responsive genes^(^^1^^,^^41^^,^^42^^)^. Activated HSP90 and HSP70 play a role in the assembling of other proteins, and they are involved in the regulation of kinetic partitioning between-folding, translocation and aggregation, as well as in immune, apoptotic and inflammatory processes^(^^43^^)^.

The effect of different fatty acids in GR activation has been described in mammals^(^^44^^,^^45^^)^. However, little is known about the effect of dietary lipids on GR transcripts in fish. Benitez-Dorta *et al.*^(^^22^^)^ showed an effect of dietary oils on GR gene expression in different tissues of Senegalese sole subjected to stress. Besides, certain fatty acids, and their metabolites, such as ARA have been shown to regulate HSP in humans^(^^46^^)^. Highly unsaturated fatty acids have been described to exert a heat shock-induced increase of HSP70 gene expression in leucocytes isolated from the pronephros of rainbow trout following incubation with DHA and ARA compared with unsupplemented cells^(^^47^^)^, whereas Benitez-Dorta *et al.*^(^^22^^)^ reported a reduced gene expression of HSP in different tissues of Senegalese sole fed vegetable oil-based diets.

Accordingly, the aim of the present study was to give insights on the effect of different fatty acids on cortisol production in ACTH-stimulated head kidney. For that purpose, European sea bass (*Dicentrarchus labrax*) isolated head kidney cells were maintained in a superfusion system and were incubated with different fatty acids before an ACTH pulse. European sea bass is highly susceptible to stressful situations and opportunistic pathogen incidence. Besides, the tolerance of this species to dietary changes such as the type of feed oil seems to be lower than that of other marine fish species such as the gilthead sea bream^(^^48^^)^.

## Materials and methods

All the experimental conditions and sampling protocols have been approved by the Animal Welfare and Bioethical Committee from the University of Las Palmas de Gran Canaria.

### Animals and experimental conditions

Sexually immature European sea bass supplied by a Spanish fish farm (ADSA, San Bartolomé de Tirajana, Canary Islands, Spain) were acclimatised in the aquaculture facilities of the University of Las Palmas de Gran Canaria (Las Palmas, Spain) for 1 month. Fish of body weight 161·3 ± 14·5 g were distributed in four 1 m^3^ fibreglass tanks in an open seawater circulation system within the acclimatisation period. Tanks were supplied with seawater at a temperature of 23·3–23·5°C and natural photoperiod (12 h light–12 h dark). Fish were fed twice per d with a commercial feed (Biomar Iberia), 6 d per week. Before the superfusion trial, fish were kept unfed during 24 h to avoid feed interference.

### Preparation and stimulation of head kidney tissue

At the end of the acclimatisation period, for each superfusion trial, two fish were randomly taken from each tank, immediately anaesthetised with 2-phenoxyethanol (1:1000, v/v) and blood was collected from the caudal vein to minimise haemorrhage when dissecting the tissue. Superfusion protocols have been described previously in our laboratory^(^^9^^,^^21^^)^. Head kidney tissue was removed from eight fish in each superfusion trial, weighed, homogenated and kept in HEPES Ringer solution (171 mm-NaCl, 2 mm-KCl, 2 mm-CaCl_2_H_2_O, 0·25 % glucose, 0·03 % bovine serum albumin, pH 7·4) as described by Rotllant *et al.*^(^^49^^)^, which was used as the perfusion medium. Then, 200 mg of head kidney homogenates were pooled and distributed in each of the eight perfusion chambers (volume: 0·2 ml) in order to obtain a homogeneous sample in each of them, being tissue from the eight fish in each chamber and trial. Each superfusion trial was conducted in triplicates (8 × 3). The system was temperature-controlled at 18°C, and the superfusion medium was pumped at a rate of 75 ml/min by a Masterplex L/SR multichannel peristaltic pump (Cole Parmer Instrument Company), as previously described by Ganga *et al.*^(^^9^^)^.

After a stabilisation period of 180 min required for cortisol to reach a stable baseline level as previously described^(^^9^^,^^21^^,^^50^^)^, tissues were stimulated with ACTH at a concentration of 5 nm-hACTH_1–39_ (Sigma) for 20 min. Afterwards, superfusion was maintained, being whole pooled tissues and the supernatant fraction collected at before and after 60 min of fatty acid addition (see below), and 20, 40, 60, 110, 160 and 250 min after ACTH stimulation.

The whole pooled head kidney tissues used in each perfusion chambers from each sampling point were stored in RNA*later* (Sigma) for 8 h at 4°C. After that, RNA*later* was removed and tissues were kept at –80°C until its analysis. Samples were sent to the Department of Biotechnology and Molecular Sciences of the University of Insubria (Varese, Italy) for gene expression analysis. Besides, the supernatant fraction from each sampling point was kept at –20°C. Samples were sent to the Physiology and Cell Biology laboratory at the Universitat Autonoma de Barcelona (Barcenola, Spain) for cortisol analysis.

### Perfusion fatty acid treatments

Five treatments were carried out within the present study (plus a control one without fatty acid addition), using different fatty acids: EPA (EPA treatment), DHA (DHA treatment), ARA (ARA treatment), LA (LA treatment) and ALA (ALA treatment). These treatments were similar to the superfusion control protocol except that after the stabilisation period and before ACTH stimulation, tissues were incubated for 1 h with fatty acids diluted in less than 0·5 % of ethanol–medium (v/v) at a concentration of 50 µm, as described previously^(^^21^^)^. Triplicates were conducted for each fatty acid assayed plus the control.

### Cortisol measurements

For each fatty acid treatment plus the control (no fatty acid treatment), cortisol concentration in the supernatant fraction was determined by RIA^(^^50^^)^. The antibody used for the assay was purchased from Biolink S.L. in a final dilution of 1:6000. This antibody cross-reactivity is 100 % with cortisol, 11·40 % with 21-desoxycorticosterone, 8·90 % with 11-desoxycortisol and 1·60 % with 17α-hydroxyprogesterone. Radioactivity was quantified using a liquid scintillation counter. Cortisol levels are given as ng/g tissue per h, as previously described by Ganga *et al.*^(^^21^^)^.

### Preparation of total RNA

Total RNA was extracted from all the samples using PureYield RNA Midiprep System (Promega), following the protocol described in the PureYield™ RNA Midiprep System Technical Manual no. TM279 (available online at: www.promega.com/tbs).

The quantity of the extracted RNA was calculated using the absorbance at 260 nm, whereas the integrity of RNA was assessed by agarose gel electrophoresis. Crisp 18S and 28S bands, detected by ethidium bromide staining, were indicators of the intact RNA.

### Quantitative real-time RT-PCR

#### Generation of in vitro-transcribed mRNA for standard curves

The approach used for the real-time quantification of our target genes expression relied on the standard curve method for target mRNA quantification. The target genes were c-*fos*, StAR, CYP11β and HSP70. Following this method, the number of each gene transcript copies could be quantified by comparing them with a standard graph constructed using the known copy number of mRNA of each target gene. The first step in this direction is the generation of standards of mRNA by *in vitro* transcription. As an example, in the case of c-*fos*, a forward and a reverse primer were designed based on the mRNA sequences of *D. labrax* c-*fos* that we have previously identified^(^^38^^)^ (Genebank accession no. DQ838581). This primer pair was used to create templates for the *in vitro* transcription of mRNA for c-*fos*. The forward primer was engineered to contain a T3 phage polymerase promoter gene sequence to its 5′ end (5′-*caattaaccctcactaaaggg*TCTCACAGAGCTCACCCCTA-3′) and used together with the reverse primer (5′-TGGTCTCCATTACTCCTTCCC-3′) in a conventional RT-PCR of total sea bass head kidney RNA. RT-PCR products were then checked on a 2·5 % agarose gel stained with ethidium bromide, cloned using the pGEM^®^-T cloning vector system (Promega) and subsequently sequenced in the SP6 direction.

*In vitro* transcription was performed using T3 RNA polymerase and other reagents supplied in the Promega RiboProbe *In Vitro* Transcription System kit according to the manufacturer's protocol.

The molecular weight (MW) of the *in vitro*-transcribed RNA for c-*fos* was calculated according to the following formula: 
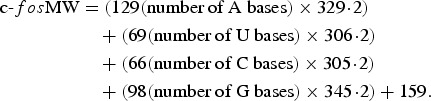


The result was 126 182·2. Spectrophotometry at 260 nm gave a concentration of 132·8 ng/μl for c-*fos*. Therefore, the concentration of the final working solution was 6·34 × 10^11^ molecules/μl.

The same aforementioned approach was used for the *in vitro* transcription of the other target genes such as StAR, CYP11β, GR, HSP90 and HSP70. The primers used are shown in Table 1.

**Table 1.** Sequences of primers used to synthesise standard mRNA

Gene	Symbol	Accession no.	Primer sequence (5′–3′)
Steroidogenic acute regulatory protein	StAR	EF409994	Forward: *gtaatacgactcactataggg*ACTCAGCACCCGAAAATGC
			Reverse: ACTTTGCCAACCACCTCAG
Cytochrome P450 11β	CYP11 β	AF449173	Forward: *gtaatacgactcactatagggCTCAAGAACGGTGAGGAGTGG*
			Reverse: CTTCTCCTCATCTCCCTCCA
Glucocorticoid receptor	GR	AY549305	Forward: *gtaatacgactcactatagggGCCTTTTGGCATGTACTCAAACC*
			Reverse: GAACAGGTATGGAGAGTCGTCCC
Heat shock protein 90	HSP90	AY395632	Forward: *gtaatacgactcactataggg*CCAACGACTGGGAGGATCAC
			Reverse: GAGTTCCGGGCCCTGC
Heat shock protein 70	HSP70	AY423555	Forward: caattaaccctcactaaagggCCATCCTGACCATCGAAGAC
			Reverse: TTGTCCATCTTGGCGTCAC

The MW of the *in vitro*-transcribed RNA calculated according to the aforementioned formula were 117 433·8 for HSP70, 73 451·4 for StAR and 96 414·6 for CYP11. Spectrophotometry at 260 nm gave a concentration of 33·7 ng/μl for HSP70; 201·1 for CYP11b and 104·0 for StAR. Therefore, the concentrations of the final working solutions were 1·73 × 10^11^ molecules/μl for HSP70, 1·26 × 10^12^ for CYP11b and 8·53 × 10^11^ molecules/μl for StAR.

#### Generation of standard curves for stress-related genes

The mRNA of target genes produced by *in vitro* transcription were used as quantitative standards in the analysis of experimental samples. Defined amounts of mRNA of each gene, at 10-fold dilutions, were subjected to real-time PCR using One-Step TaqMan EZ RT-PCR Core Reagents (Life Technologies), including 1 × Taqman buffer, 3 mm-MnOAc, 0·3 mm-dNTP except dTTP, 0·6 mm-dUTP, 0·3 µm forward primer, 0·3 µm reverse primer, 0·2 µm FAM-6 (6-carboxyfluorescein-labelled probe), 5 units *rTH* DNA polymerase and 0·5 units AmpErase UNG enzyme in a 30 µl reaction. RT-PCR conditions were: 2 min at 50°C, 30 min at 60°C, and 5 min at 95°C, followed by forty cycles consisting of 20 s at 92°C, 1 min at 62°C. The cycle threshold (C_T_) values obtained by amplification were used to create standard curves for target genes.

#### Quantification of transcripts by One-Step TaqMan real-time RT-PCR

Total RNA (100 ng) extracted from the experimental samples was subjected, in parallel to 10-fold-diluted, defined amounts of standard mRNA, to real-time PCR under the same experimental conditions as for the establishment of the standard curves. Real-time Assays-by-Design^SM^ PCR primers and gene-specific fluorogenic probes were designed by Life Technologies. Primer sequences and Taqman® probes of the four target genes are shown in Table 2.

**Table 2.** Primers and probes for quantitative real-time PCR

Gene	Symbol	Nucleotide sequence (5′–3′)	One step Taqman real-time standard curve quality
		Forward: CAGCAAAATGCCGCAACAG	*R*^2^ 0·995
c-*fos*	c-*fos*	Reverse: TGGACTTCTCATCCTCTAGCTGATC	Efficiency 89·45 %
		Taqman probe: GAGCTTACAGACACTCTG	
		Forward: AGCGGAGAATGGACCTACCT	*R*^2^ 0·982
Steroidogenic acute regulatory protein	StAR	Reverse: GAAGACCCAAATAAGACCAAGTTCAC	Efficiency 90·379 %
		Taqman probe: ATAGTCATGAAGCCCTGTG	
		Forward: CTTCGGCAGTAAAGTGCTTTCTAC	*R*^2^ 0·993
Cytochrome P450 11β	CYP11b	Reverse: GGATTTCTGTCGAATGCTGCG	Efficiency 82·47 %
		Taqman probe: GCTTGATGAGGTGGCGA	
		Fw: GGACATCAGCCAGAACAAGAGA	*R*^2^ 0·996
Heat shock protein 70	HSP70	Reverse: GAGAACCCTGTCCTCCAGC	Efficiency 99·276 %
		Taqman probe: GCTTGTGAGAGGGCCAA	
		Forward: GCCTTTTGGCATGTACTCAAACC	*R*^2^ 0·997
Glucocorticoid receptor	GR	Reverse: GAACAGGTATGGAGAGTCGTCC	Efficiency 94·417 %
		Taqman probe: GTGGTTGGGGAGAGCTG	
		Forward: CCAACGACTGGGAGGATCAC	*R*^2^ 0·998
Heat shock protein 90	HSP90	Reverse: GAGTTCCGGGCCCTGC	Efficiency 86·23 %
		Taqman probe: CTGTCAAGCACTTCTCG	

TaqMan® PCR was performed using the StepOne Real-time PCR System (Life Technologies). To reduce pipetting errors, master mixes were prepared to set up duplicate reactions (2 × 30 µl) for each sample.

#### Sample quantification

Data from Taqman® PCR runs were collected with the StepOne Real Time Sequence Detector Program. C_T_ values corresponded to the number of cycles at which the fluorescence emission monitored in real time exceeded the threshold limit. The C_T_ values were used to create standard curves to serve as a basis for calculating the absolute amounts of mRNA in total RNA.

### Calculation and statistical analysis

Quantitative PCR data were analysed by one-way ANOVA and each time point was analysed separately. A *post hoc* test was applied (Tukey). We used the statistics package SPSS Statistics 21 (IBM). The other data were statistically compared using one-way ANOVA. The level of statistical significance was set at *P* < 0·05.

## Results

### Cortisol released from superfused head kidney

**Fig. 1.**
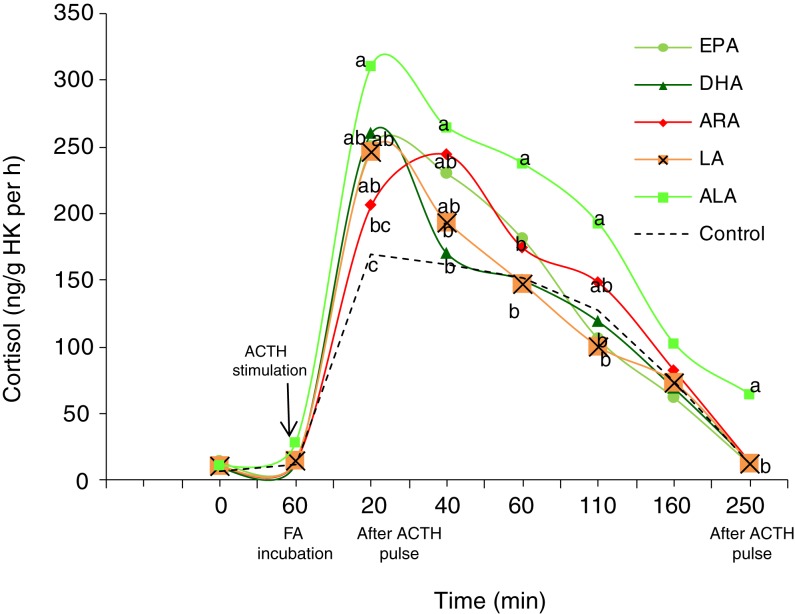
Absolute cortisol secretion by European sea bass (*Dicentrarchus labrax*) head kidney (HK) after adrenocorticotrophin hormone (ACTH) stimulation following incubation with highly unsaturated fatty acids (FA): EPA, DHA, arachidonic acid (ARA), linoleic acid (LA) and α-linolenic acid (ALA). ^a,b,c^ Different letters for a given time indicate significant differences (*P* < 0·05).

Basal cortisol values were obtained after the stabilisation period (180 min) and no significant differences were found among values of different fatty acid treatments. After 1 h of incubation with fatty acid, the release of cortisol remained low (Fig. 1). After ACTH stimulation, cortisol values increased in all the experimental groups, the values obtained for head kidney from the EPA, DHA, LA and ALA treatments being significantly higher (*P* < 0·05) when compared with the control group after 20 min of ACTH stimulation. At 40 min after ACTH stimulation, cortisol values of ALA, ARA and EPA treatments were significantly higher (*P* < 0·05) that the control values. After 60 min of ACTH stimulation values of cortisol of head kidney from the ALA treatment showed the highest values, within all the superfusion trials, being significantly (*P* < 0·05) higher for all the sampling points except for 160 min after ACTH stimulation.

### StAR, c-fos, CYP11b, GR, HSP70 and HSP90 mRNA copy number in sea bass anterior kidney cells during the perfusion trial

**Fig. 2.**
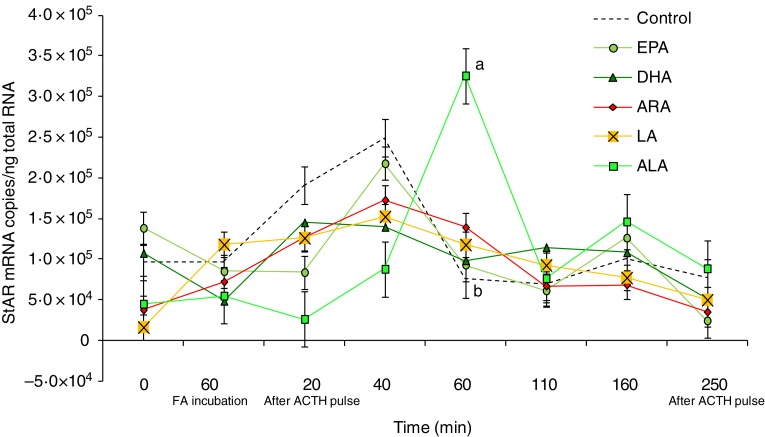
Expression levels of the steroidogenic acute regulatory protein (StAR) gene measured by real-time PCR in *Dicentrarchus labrax* head kidney cells in the course of the perfusion trial. StAR mRNA copy number was normalised as a ratio to 100 ng total RNA. Cells were sampled after the stabilisation period (0 h), 60 min after highly unsaturated fatty acids (FA) incubation (EPA, DHA, arachidonic acid (ARA), linoleic acid (LA) and α-linolenic acid (ALA)), 20 min after adrenocorticotrophin hormone (ACTH) stimulation, and then sequentially at 40, 60, 110, 160 and 250 min following the ACTH pulse. The means of three replicates in each sampling point are shown. Bars indicate standard error of the mean. ^a,b^ Different letters for a given time indicate significant differences (*P* < 0·05).

The mRNA copies of StAR were significantly (*P* < 0·01) affected by the type of fatty acid used in the perfusion trial (Fig. 2), with ALA inducing an increase in expression after 60 min of ACTH pulse. The expression level of this group at this time point of perfusion trial was double that obtained by using the other fatty acids.

**Fig. 3.**
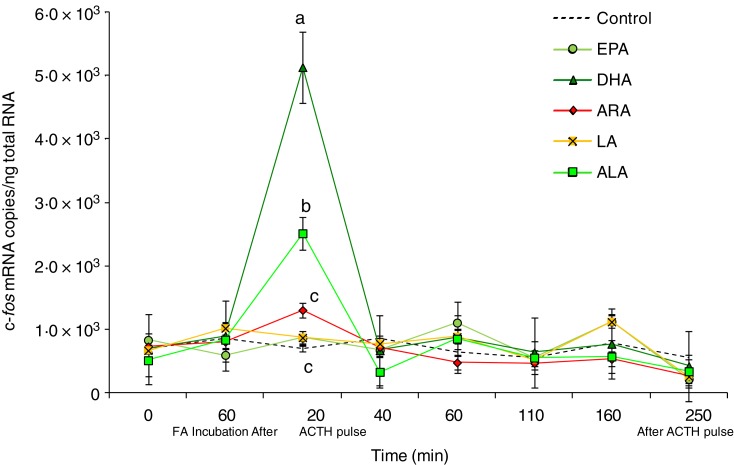
Expression levels of the c-*fos* gene measured by real-time PCR in *Dicentrarchus labrax* head kidney cells in the course of the perfusion trial. c-*fos* mRNA copy number was normalised as a ratio to 100 ng total RNA. Cells were sampled after the stabilisation period (0 h), 60 min after highly unsaturated fatty acids (FA) incubation (EPA, DHA, arachidonic acid (ARA), linoleic acid (LA) and α-linolenic acid (ALA)), 20 min after adrenocorticotrophin hormone (ACTH) stimulation, and then sequentially at 40, 60, 110, 160 and 250 min following the ACTH pulse. The means of three replicates in each sampling point are shown. Bars indicate standard error of the mean. Differences were determined by one-way ANOVA and each time point was analysed separately. A *post hoc* test was applied (Tukey). ^a,b,c^ Different letters indicate significantly different means from controls, for the time point tested (*P* < 0·01).

C-*fos* mRNA in sea bass head kidney cells in response to the perfusion trial are presented in Fig. 3. As shown, incubation for 60 min with DHA, ALA and ARA contributed to a significant increase in c-*fos* transcripts (*P* < 0·01) after 20 min of ACTH induction, as compared with the controls. DHA was the fatty acid that induced the highest c-*fos* level of expression with 5·13 × 10^5^ mRNA copy number/ng total RNA, followed in a decreasing pattern by ALA with 2·51 × 10^3^, and ARA with 1·31 × 10^3^. The same time of incubation did not have an effect on c-*fos* transcript levels in cells incubated with either EPA or LA. Indeed, in these cells the mRNA copy number was the same as that of the controls. Subsequently, the expression levels of c-*fos* in different treatment groups fluctuated insignificantly as compared with the control values until the end of the perfusion trial (Fig. 3).

**Fig. 4.**
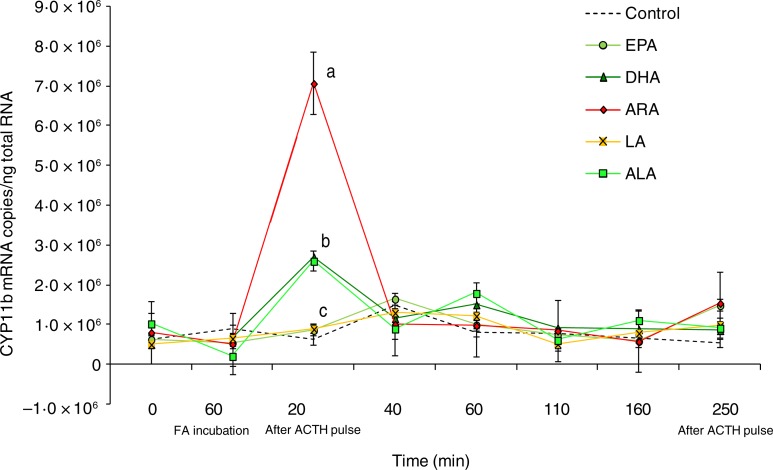
Expression levels of the cytochrome P450 11β (CYP11b) gene measured by real-time PCR in *Dicentrarchus labrax* head kidney cells in the course of the perfusion trial. CYP11b mRNA copy number was normalised as a ratio to 100 ng total RNA. Cells were sampled after the stabilisation period (0 h), 60 min after highly unsaturated fatty acids (FA) incubation (EPA, DHA, arachidonic acid (ARA), linoleic acid (LA) and α-linolenic acid (ALA)), 20 min after adrenocorticotrophin hormone (ACTH) stimulation, and then sequentially at 40, 60, 110, 160 and 250 min following the ACTH pulse. The means of three replicates in each sampling point are shown. Bars indicate standard error of the mean. Differences were determined by one-way ANOVA and each time point was analysed separately. A *post hoc* test was applied (Tukey). ^a,b,c^ Different letters indicate significantly different means from controls, for the time point tested (*P* < 0·01).

There were significant effects (*P* < 0·01) of the type of fatty acid used during the perfusion trial on CYP11b gene expression (Fig. 4). ARA, ALA and DHA treatments at 20 min after ACTH stimulation reached values up to 7·0 × 10^6^ for ARA and 2·8 × 10^6^ for ALA and DHA in comparison with 0·8 × 10^6^ mRNA copies for the EPA, LA and control experimental groups (Fig. 4).

**Fig. 5.**
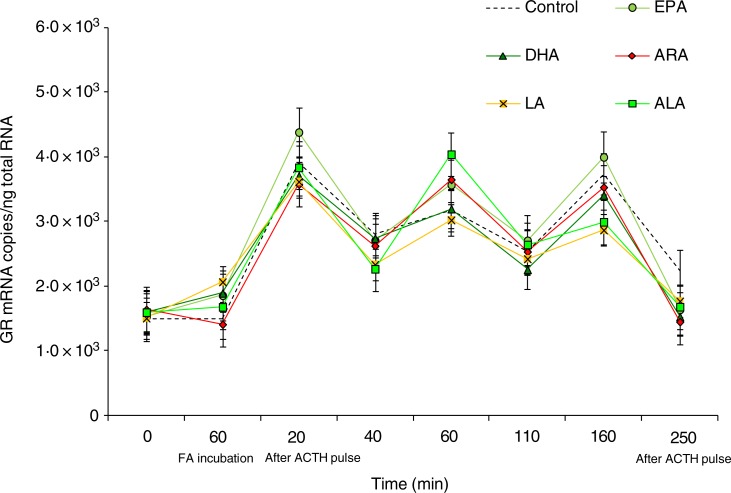
Expression levels of the glucocorticoid receptor (GR) gene measured by real-time PCR in *Dicentrarchus labrax* head kidney cells in the course of the perfusion trial. GR mRNA copy number was normalised as a ratio to 100 ng total RNA. Cells were sampled after the stabilisation period (0 h), 60 min after highly unsaturated fatty acids (FA) incubation (EPA, DHA, arachidonic acid (ARA), linoleic acid (LA) and α-linolenic acid (ALA)), 20 min after adrenocorticotrophin hormone (ACTH) stimulation, and then sequentially at 40, 60, 110, 160 and 250 min following the ACTH pulse. The means of three replicates in each sampling point are shown. Bars indicate standard error of the mean.

**Fig. 6.**
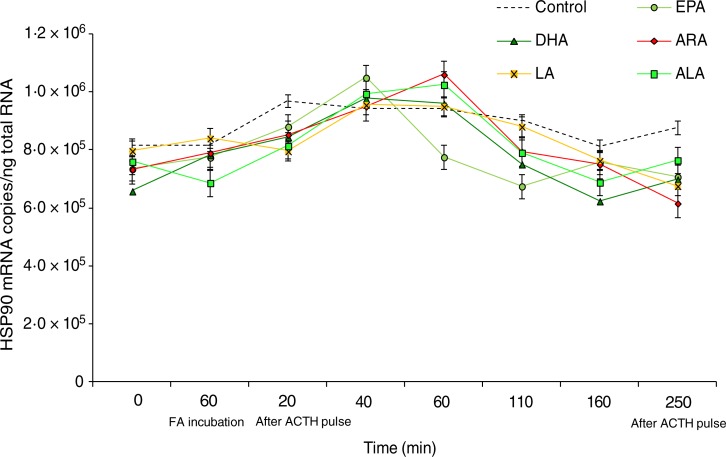
Expression levels of the heat shock protein 90 (HSP90) gene measured by real-time PCR in *Dicentrarchus labrax* head kidney cells in the course of the perfusion trial. HSP90 mRNA copy number was normalised as a ratio to 100 ng total RNA. Cells were sampled after the stabilisation period (0 h), 60 min after highly unsaturated fatty acids (FA) incubation (EPA, DHA, arachidonic acid (ARA), linoleic acid (LA) and α-linolenic acid (ALA)), 20 min after adrenocorticotrophin hormone (ACTH) stimulation, and then sequentially at 40, 60, 110, 160 and 250 min following the ACTH pulse. The means of three replicates in each sampling point are shown. Bars indicate standard error of the mean.

**Fig. 7.**
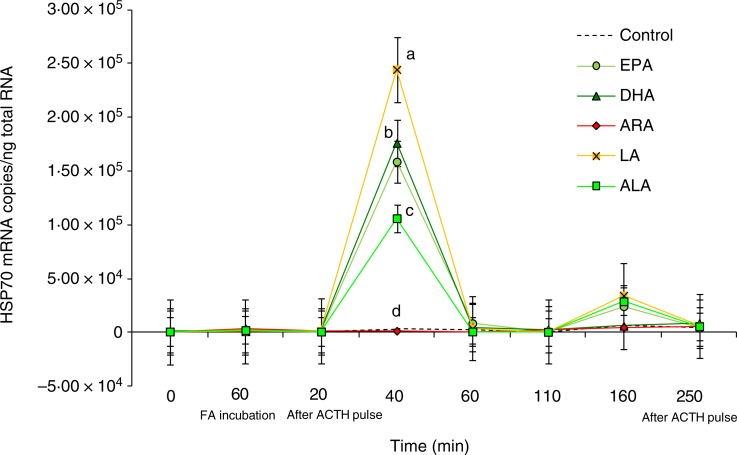
Expression levels of the heat shock protein 70 (HSP70) gene measured by real-time PCR in *Dicentrarchus labrax* head kidney cells in the course of the perfusion trial. HSP70 mRNA copy number was normalised as a ratio to 100 ng total RNA. Cells were sampled after the stabilisation period (0 h), 60 min after highly unsaturated fatty acids (FA) incubation (EPA, DHA, arachidonic acid (ARA), linoleic acid (LA) and α-linolenic acid (ALA)), 20 min after adrenocorticotrophin hormone (ACTH) stimulation, and then sequentially at 40, 60, 110, 160 and 250 min following the ACTH pulse. The means of three replicates in each sampling point are shown. Bars indicate standard error of the mean. Differences were determined by one-way ANOVA and each time point was analysed separately. A *post hoc* test was applied (Tukey). ^a,b,c,d^Different letters indicate significantly different means from controls, for the time point tested (*P* < 0·01).

We did not found any effects of the type of fatty acid used within the perfusion trial on the expression of GR or HSP90 genes (Figs. 5 and 6), whereas ACTH stimulation was associated with a significant increase in HSP70 transcripts (Fig. 7). Indeed, at 40 min after the ACTH pulse, the HSP70 mRNA copies in cells incubated with LA, DHA, EPA and ALA were significantly higher than that of the controls (*P* < 0·05), whereas the number of transcripts in cells incubated with ARA remained at the same sampling time point equal to that of the controls. At 40 min after the ACTH pulse, LA incubation induced the highest expression of HSP70 with 2·45 × 10^5^ mRNA copies/ng total RNA, followed in a decreasing pattern by DHA with 1·76 × 10^5^, EPA with 1·59 × 10^5^, and ALA with 1·06 × 10^5^ copies/ng total RNA. At 60 min after the ACTH pulse, the expression levels of HSP70 in the LA, DHA, EPA and ALA groups decreased significantly as compared with the previous sampling point (40 min), and then fluctuated insignificantly as compared with the control values till the end of the perfusion trial (Fig. 7).

The transcript copies of oxidative stress-related genes catalase (CAT), superoxide dismutase (SOD) and glutathione peroxidase (GPX) in different groups at time zero, after incubation for 60 min with different fatty acids, and at different time points after ACTH pulse did not show any significant differences from the controls (data not shown).

## Discussion

Results obtained in the present study corroborate the effect of different fatty acids on the release of cortisol in marine fish, previously described both *in vivo*^(^^6^^,^^8^^,^^16^^–^^19^^,^^51^^,^^52^^)^ and *in vitro*^(^^9^^,^^21^^)^. In agreement with the results previously obtained in gilthead sea bream under similar superfusion conditions^(^^21^^)^, both EPA and ARA induced an increase of cortisol release in ACTH-stimulated inter-renal cells of European sea bass. Moreover, the effect obtained by EPA incubation was faster in time (significantly different at 20 min after ACTH stimulation) than in the case of ARA, which showed a significant difference from the controls at 40 min after ACTH stimulation. Dietary ARA has been shown to affect whole-body cortisol and stress response in different marine fish larvae^(^^17^^,^^18^^,^^20^^,^^52^^,^^53^^)^ including European sea bass^(^^54^^)^. However, since these studies were conducted in whole larvae, and ARA was supplied through either artemia or microdiets, the assignment of an ARA direct effect on whole-body cortisol of larvae is too premature^(^^20^^)^.

The addition of DHA induced an increase of cortisol release at only 20 min after ACTH stimulation, but incubation with ALA induced an increase of cortisol release from ACTH-stimulated inter-renal cells during the entire period studied. These results also corroborate those previously reported for other marine species such as gilthead sea bream^(^^8^^,^^9^^)^ fed on diets based on linseed oil, which is an ALA-rich vegetable oil. Similarly, in Atlantic salmon smolts, feeding high-*n*-3/*n*-6 diets increased post-stress plasma cortisol^(^^7^^)^. In agreement, in human subjects, some reports indicated that increasing the dietary *n*-6:*n*-3 fatty acids ratio by increasing the ratio between LA and LNA up to 4:1 reduced blood cortisol and cholesterol levels^(^^55^^)^.

The role of different fatty acids as modulators of steroidogenesis in mammals has been widely described, most studies being related to reproductive tissues^(^^23^^,^^24^^,^^56^^,^^57^^)^ and, to a lesser extent, to the adrenal gland^(^^3^^,^^27^^)^. Steroidogenesis is modulated by a multiple range of signalling pathways, in a very complex manner^(^^3^^)^. The activation of the cAMP/PKA signalling cascade leads to the phosphorylation of transcriptional factors that regulate StAR gene transcription^(^^58^^)^, but cAMP also induces ARA release^(^^59^^)^, ARA metabolic derivatives transducing signals to the nucleus to regulate StAR gene expression, being both pathways necessary for trophic hormone-stimulated steroidogenesis^(^^60^^)^. In the present study, there were no significant differences in the ACTH-induced expression of the StAR gene immediately after the ACTH pulse, even when an increase in cortisol was detected in all experimental groups after stimulation with the trophic hormone. An increase in whole-body cortisol after stress without an increase in StAR gene expression has also been described in Senegalese sole post-larvae fed different ARA levels in the diet^(^^20^^)^. Those authors hypothesised that higher StAR transcription may not be necessary for cortisol production. StAR is involved in the transport of cholesterol through the mitochondrial membrane of the steroidogenic cells to be used as the substrate for steroid synthesis. Such transport may also have been carried out by an available pool of inactive StAR protein^(^^61^^)^. However, Stocco *et al.*^(^^3^^)^ pointed out that a chronic response involves an increase in the transcription/translation of steroidogenic-related genes whereas during an acute response to hormonal stimulation there is an absolute requirement for *de novo* protein synthesis for an acute production of steroids. Thus, it is also probable that the maximum peak of StAR expression is produced earlier than the sampling time point of 20 min after the ACTH pulse, explaining the lack of response of this gene found in our experiment and other previous studies^(^^20^^)^. It is interesting to point out that within the present study, a significant increase of StAR gene expression in the ALA treatment was found after 60 min of ACTH stimulation. The expression of StAR is directly related to the activity of the PG endoperoxide synthase II (PTGS2), which in turn is modulated by PUFA^(^^62^^)^. α-Linolenate, derived from ALA, is a poor substrate for PTGS2 in comparison with arachidonate or linoleate^(^^63^^)^. Thus the activity of PTGS2 is expected to be lower in an ALA-enriched medium, and a putative stimulatory effect on steroidogenesis through PTGS2 inhibition may have contributed to an increase of StAR gene expression. This could explain the increase of cortisol release found in ALA treatment after ACTH stimulation in the present study and how linseed oil, which is rich in ALA, increases plasma cortisol in marine fish^(^^6^^,^^8^^,^^22^^)^.

Steroidogenesis has been described as being linked to the action of different ARA metabolites. The critical role of ARA-mediated metabolites in steroidogenesis has been widely described in mammals, via activation of secretory phospholipase A2 (PLA2) through activation of G protein after ACTH stimulation^(^^3^^)^. PLA2 catalyses the release of fatty acids from phospholipids. Alves Martins *et al.*^(^^20^^)^ have proposed a direct relationship between expression of PLA2 and whole post-larvae cortisol in Senegalese sole after 3 h of stress. However, other factors must be taken into consideration to elucidate this relationship, including the fast secretory PLA2-induced release of ARA after trophic hormone stimulation (less than 1 min)^(^^3^^)^ since most of the previous studies evaluated PLA2 after several minutes or even hours. Different types of secretory PLA2, such as group X secretory PLA2, have been described to reduce StAR gene expression in mouse adrenals^(^^64^^)^. Besides, at least one other ARA-releasing pathway has been described in steroidogenesis, which depends on the cAMP-induced activation of the CoA thioesterase^(^^65^^)^. It remains to be determined whether steroidogenesis dependence of secretory PLA2 plays a similar functional role in fish after stress.

The fatty acids released are metabolised through one of the three enzymic pathways: COX-2, LOX or epoxygenase. It has been reported that ARA metabolites produced through the LOX pathway stimulated steroidogenesis in Leydig cells of mammals^(^^23^^)^, whereas COX-2 appears to be responsible for a tonic inhibition of steroidogenesis in those cells^(^^25^^)^. ARA metabolites produced by epoxygenase activity, the epoxyeicosatrienoic acids, regulate StAR at the transcription level^(^^25^^)^. PG modulate the sensitivity of the mammalian hypothalamus–pituitary–inter-renal axis, altering the stress response^(^^66^^)^, whereas COX-derived PG have been shown to increase *in vitro* cortisol release in inter-renal tissue of female frogs^(^^67^^)^.

As far as we know, there are no studies in fish on the effect of the different ARA metabolites in steroidogenesis, although the implication of COX-derived metabolites in cortisol release has been suggested in fish^(^^17^^–^^19^^,^^51^^,^^68^^)^. Ganga *et al.*^(^^21^^)^ demonstrated that the role of ARA and EPA as modulators of the release of cortisol from ACTH-stimulated inter-renal cells is mediated, at least in part, by COX-2-derived metabolites, since the incubation of head kidney in a indomethacin-enriched medium decreased the release of cortisol from gilthead sea bream inter-renal cells incubated in an ARA- or EPA-enriched medium^(^^21^^)^. In a similar way, the effect of linseed oil in sea bream diets on the *in vitro* release of cortisol from inter-renal cells after ACTH stimulation has been proved to be mediated also by COX-2- and LOX-derived metabolites^(^^9^^)^.

Although the role of ARA and EPA on steroidogenesis seems to be mainly mediated by their role in eicosanoid production, Ganga *et al.*^(^^21^^)^ suggested other mechanisms for the role of DHA in the release of cortisol from inter-renal cells in sea bream. The utilisation of indomethacin, an inhibitor of COX activity, did not affect the release of cortisol from head kidney of sea bream in a DHA-enriched medium. DHA has been described to reduce PGF2-α, a PG which has been proved to modulate the expression of StAR^(^^69^^)^. DHA can also modulate steroidogenesis through its role as a PPAR-α activator^(^^70^^)^ and steroidogenic factor 1 (SF-1)^(^^71^^,^^72^^)^, that in turn modulate genes involved in the stress response such as StAR, among others^(^^73^^)^. DHA can also influence steroidogenesis through its role as a regulator of intracellular Ca^(^^74^^)^. The increase in intracellular Ca^2+^, either released from intracellular stores or by mobilisation from extracellular spaces, is known to play an important role in steroidogenesis^(^^3^^)^.

In the present study, there was a clear effect of DHA on the expression of c-*fos*. C-*fos* expression significantly increased 5-fold after 20 min of stress when compared with control. C-*fos* is a member of the AP-1 response elements^(^^75^^)^. The role of AP-1 response elements on steroidogenesis has been described in mammals. In the adult rat, it has been proved that c-*fos* mRNA and FOS protein are reliable indices of adrenocortical activation^(^^76^^)^. C-*fos* overexpression in Y1 adrenal cells led to a decrease in StAR gene promoter activity^(^^29^^)^, whereas in contrast, overexpression of c-*fos* enhanced steroidogenesis in MA-10 cells by increasing StAR gene expression and interaction with transcription factors such as SF-1 to regulate steroidogenesis^(^^77^^)^, acting as an endogenous promoter^(^^78^^)^. The role of c-*fos* in steroidogenesis has been proved to be cAMP activation independent^(^^78^^)^, suggesting the possible action of DHA on steroidogenesis through mechanisms other than the COX-2 pathway, as pointed out by Ganga *et al.*^(^^21^^)^. As far as we know, this is the first time that c-*fos* expression has been related to the stress response and fatty acids in European sea bass and even fish. The clear effect found for the DHA treatment together with the increase also seen in the ARA and ALA treatments indicate different ways of modulation of cortisol release from the inter-renal cells of marine fish. Further studies must be conducted to clarify the specific role of c-*fos in vivo* when feeding marine fish with diets containing different type of oils.

Fatty acids may affect not only cholesterol transportation through the mitochondrial membrane, but also through the metabolic pathway of cholesterol to produce steroids. Interestingly, oxidative stress has been shown to alter steroidogenesis by producing inappropriate StAR-mediated trafficking of peroxidised cholesterol in streroidogenic tissues, resulting in damage and dysfunction in mitochondria^(^^79^^)^. In the present study, all the indicators of oxidative stress measured remained unaltered within all the experiment, values obtained for each treatment being equal to the control values, and thus any possible effect of oxidative-induced metabolites of any of the fatty acid used within the study can be rejected.

There are few data on how fatty acids modulate the expression of cytochromes related to cortisol synthesis, the so-called CYP11, that have been described in several fish species^(^^80^^)^ including European sea bass^(^^81^^)^. As far as we know, there are no previous studies on the 11β-hydroxylase (CYP11b) mRNA of European sea bass. Aluru & Vijayan^(^^82^^)^ reported an increase of 11β-hydroxylase mRNA abundance together with plasma cortisol concentration, in groups of rainbow trout in response to 1 h of handling, and preceded the rise of cortisol level in zebrafish (*Danio rerio*) embryos^(^^83^^)^, establishing that CYP11β expression reacts to stressful stimuli to synthesise cortisol. Although no evidence has been found on the role of dietary fatty acids on the expression of cytochrome genes in fish, it has been demonstrated that the gene expression of 17α-hydroxylase, a member of cytochrome P450 which participates in the cortisol pathway, is down-regulated in mice fed with a high-fish oil diet^(^^84^^)^. Within the present study we found an increase (7-fold) in CYP11b gene expression in head kidney from the ARA treatment after 20 min of ACTH stimulation. Besides, head kidney from both the DHA and ALA treatments also showed an increased (2-fold) gene expression in CYP11b when compared with the control group. These results correspond to those groups in which c-*fos* expression is also increased. More studies are required to obtain information *in vitro* and *in vivo* on the role of fatty acids in the expression of the different CYP, since not only CYP11b determines steroidogenesis^(^^80^^)^.

Finally, within the present study, no effects of the different fatty acid used were found on glucocorticoid receptor gene expression. There is not so much information on the role of fatty acids as modulators of GR expression. Feeding soyabean oil has been found to affect glucocorticoid receptors in mice^(^^45^^)^, whereas Benitez-Dorta *et al.*^(^^22^^)^ found a marked effect of the type of dietary oils on the expression of GR genes in different tissues of Senegalese sole. Those authors found a reduction in the stress-induced increase of liver GR genes in Senegalese sole fed a vegetable oil-based diet in comparison with fish oil-fed sole. In muscle, feeding vegetable oils, particularly soyabean oil, caused an over-expression of the GR2 gene in response to chasing stress. Besides, Benitez-Dorta *et al.*^(^^22^^)^ found that the use of dietary vegetable oils in Senegalese sole reduced the gene expression of HSP90AB in muscle and HSP70 in intestine. HSP70 gene expression has been reported to be regulated by other dietary factors such as starvation^(^^85^^)^, energy restriction^(^^86^^)^ or arginine supplementation^(^^87^^)^. PUFA, and specifically DHA and ARA, have been shown to enhance the heat-induced stress response in rainbow trout (*Oncorhynchus mykiss*) leucocytes^(^^47^^)^, whereas an increased gene expression of HSP90 has been also found in the liver of rainbow trout fed alternative diets containing soyabean meal^(^^88^^)^.

In the present experiment, no effects were detected on HSP90 gene expression, and, interestingly, the present results showed a significantly increased HSP70 gene expression in all the fatty acid treatments except the ARA treatment. ARA has been described as a potent modulator of HSP in humans^(^^46^^)^, through the activation of heat shock factor, being ARA metabolites, and specially PGE2, was more related to the HSP activation^(^^89^^)^. Jurivich *et al.*^(^^46^^)^ demonstrated that 20 µm-ARA was enough to activate HSP72 expression at 37°C in HeLa cells, but ARA concentration up to 20 µm had no effect on reported activity in the absence of heat shock^(^^89^^)^, demonstrating that the effect of ARA is dose dependent. This could be in agreement with the results obtained in the present experiment, if we consider that the exposure of head kidney to a medium enriched with ARA in the doses used in this experiment could be exceeding the concentration required for HSP gene expression activation, whereas the rest of the treatments showed an effect. It must be taken into consideration that the present study was conducted on the head kidney. The complexity of this tissue, with immune cells associated, inter-renals and other constitutive and renal tissues associated. Further experiments are required to elucidate the effect of fatty acids in the activation of HSP and GR in other target tissues for cortisol action.

In conclusion, the results obtained in the present study showed a clear modulation of different fatty acids on cortisol release from European sea bass, partly mediated by the effects on the expression of stress-related genes. Further studies are needed to elucidate the role of those fatty acids as effectors on the expression of stress-related genes *in vivo*, feeding animals different type of oils and levels of essential fatty acids in the diet. However, with the present results we corroborate previous results indicating that ALA increases basal and post-stress cortisol in marine fish, clarifying that the ALA-induced elevation of cortisol release from the inter-renal cells can be mediated by different pathways and effects on different genes. Whether the increased amount of cortisol from the ALA treatment is due to an addition of different effects remains unclear, but induction of StAR and c-*fos* clearly increased the ACTH-induced cortisol release from head kidney enriched with ALA.

As far as we know, this is the first time that c-*fos* expression has been studied in European sea bass or even in any fish species, associated with different fatty acids and the stress response. The clear results obtained by DHA treatment explains a previous hypothesis on the role of DHA as a modulator of the release of cortisol, proposed to be independent of the COX-2 pathway. The use of c-*fos* as a bioindicator of fatty acid-mediated modulation of cortisol release is still premature, but the present results indicated the potential use of this indicator in fatty acid studies. Further experiments need to be conducted *in vivo* to clarify this. The same can be proposed for the effect of ARA on the expression of CYP11b. As this cytochrome modulates the final step in the cortisol synthesis pathway, its potential use as a bioindicator of ARA-mediated stress needs to be studied *in vivo*.
